# Engineering exosomes for bone defect repair

**DOI:** 10.3389/fbioe.2022.1091360

**Published:** 2022-12-07

**Authors:** Shaoyang Ma, Yuchen Zhang, Sijia Li, Ang Li, Ye Li, Dandan Pei

**Affiliations:** Key Laboratory of Shaanxi Province for Craniofacial Precision Medicine Research, College of Stomatology, Xi’an Jiaotong University, Xi’an, Shaanxi, China

**Keywords:** bone regeneration, engineering exosomes, exosomal cargos, exosome-integrated biomaterials, osteogenesis

## Abstract

Currently, bone defect repair is still an intractable clinical problem. Numerous treatments have been performed, but their clinical results are unsatisfactory. As a key element of cell-free therapy, exosome is becoming a promising tool of bone regeneration in recent decades, because of its promoting osteogenesis and osteogenic differentiation function *in vivo* and *in vitro*. However, low yield, weak activity, inefficient targeting ability, and unpredictable side effects of natural exosomes have limited the clinical application. To overcome the weakness, various approaches have been applied to produce engineering exosomes by regulating their production and function at present. In this review, we will focus on the engineering exosomes for bone defect repair. By summarizing the exosomal cargos affecting osteogenesis, the strategies of engineering exosomes and properties of exosome-integrated biomaterials, this work will provide novel insights into exploring advanced engineering exosome-based cell-free therapy for bone defect repair.

## 1 Introduction

Bone is the central element in skeletal tissues of the human body, and provides a framework for attachment of muscles and other tissues, enables body movements, provides protection of internal organs from injury, promotes blood cells production, and balances calcium and acid/base homeostasis (Elefteriou, 2018). However, the regeneration of critical-size bone defects is still a major clinical challenge and globally costs up to $45 billion per year (Mauffrey et al., 2015; Bharadwaz and Jayasuriya, 2020). Recently, stem cell therapy is considered as a potential strategy for bone defect regeneration (Tan SHS. et al., 2020), and several clinical studies have demonstrated mesenchymal stromal/stem cells (MSCs) to be safe and efficacious for the treatment of bone defects and diseases (Liebergall et al., 2013; Chen et al., 2016; Castillo-Cardiel et al., 2017; Hernigou et al., 2018a; Hernigou et al., 2018b). Nevertheless, cellular therapies incur significant costs and challenges as they require stringently monitored manufacturing, handling, and storage to ensure optimal viability and potency of cells needed for transplantation (Tan et al., 2021). More importantly, accumulating evidence indicates that the positive effect of MSCs on tissue repair is to stimulate the activity of tissue-resident recipient cells through paracrine, such as exosomes, rather than directly differentiate into parenchymal cells to repair or replace damaged tissue (Liang et al., 2014; Zhang et al., 2016). Such concerns have driven the search for alternate therapeutic strategies and cell-free therapies based on exosomes have become strongly established in the landscape of regenerative medicine.

Exosome is a subclass of membrane-coated extracellular vesicles with sizes of 30–150 nm (Tkach and Thery, 2016). As one of the most revolutionary contributions to cell biology in the past 30 years (Wang Y. et al., 2019), exosomes can exert multiple biological functions by targeting recipient cells and inducing signaling *via* receptor-ligand interactions, endocytosis and/or phagocytosis (Bobrie et al., 2012; Colombo et al., 2014; Hoshino et al., 2015; Shang et al., 2021). Exosomes have been experimented with many animal models for the regeneration of bone, osteochondral, and cartilage injury/diseases such as osteoarthritis (OA), osteoporosis, osteonecrosis, and inflammatory bone loss in periodontitis with enhanced tissue formation and integration (Kuang et al., 2019; Kim et al., 2020; Lei et al., 2022). Furthermore, several exosome-based clinical experiments of orthopedic diseases have been performed based on US-NIH clinical trial database (https://clinicaltrials.gov/). However, there still are several constraints to exosome clinical applications for bone defect repair: 1) unclear mechanism of promoting bone tissue regeneration; 2) poor retention and targeting ability of exosome at the bone defect site; 3) low extraction rate and complex separation process.

In view of the shortcomings of natural exosomes, a growing number of studies are aiming to develop engineering exosomes based on modifying exosomal cargos or/and incorporating biomaterials (Bei et al., 2021; Lathwal et al., 2021; Liang et al., 2021). Here we will review the recent research of engineering exosome used in bone defect repair, and highlight the bioactive cargos and construction strategies. Additionally, we will also summarize the application of biomaterials to impregnate exosome and focus on how the properties of biomaterials assist exosome to promote bone regeneration. By reviewing currently available knowledge, this present review will contribute to the clinical knowledge and may have implications for the engineering design of exosomes used in bone defect repair.

## 2 Osteogenic cargos in exosomes

**FIGURE 1:**
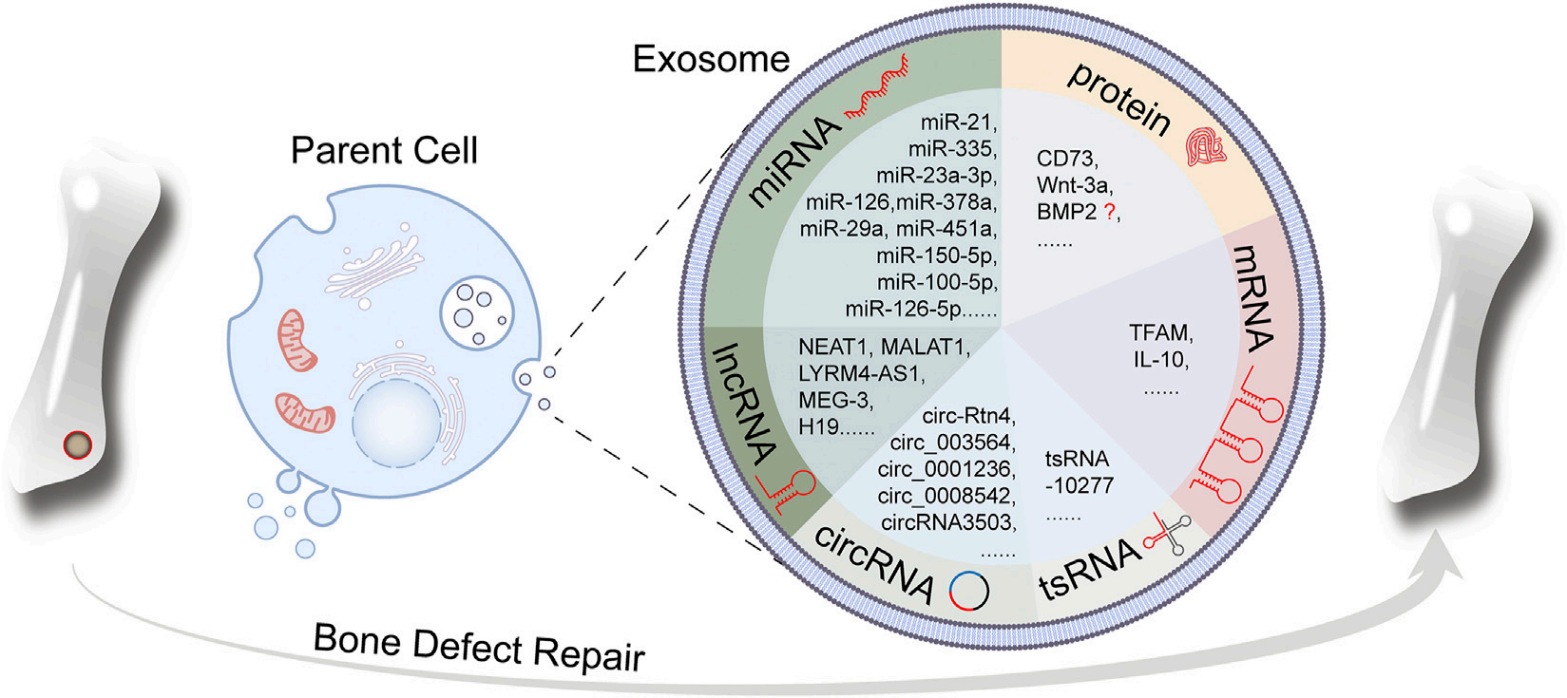
Schematic of exosomal cargos (miRNA, lncRNA, circRNA, tsRNA, mRNA and protein) with the function of promoting bone regeneration.

In the past decade, numerous exosomal bioactive cargos have been revealed (Kalluri and LeBleu, 2020). Exosomal cargos are dependent on the parent cell type and vary between different physiological or pathological conditions (Meng et al., 2019). The vesicular structure of exosome provides an enclosed space to protect exosomal cargos against degradation. In return, exosomal cargos are the foundation to endow exosomes with various biological functions. In this section we will review recent research about exosomes in bone regeneration and focus on the functions of exosomal cargos and their molecular mechanisms (Figure 1).

### 2.1 Non-Coding RNA

Non-coding RNAs (ncRNAs) refer to the RNAs that lack protein-coding regions, and have the potential to regulate gene expression at transcriptional, post-transcriptional, and translational levels, thereby modulating associated signaling networks (Bhat et al., 2020). NcRNAs have become a hot topic of increasing concern after the completion of the Human Genome Project (Lander et al., 2001), which showed only 1.2% of genes in the genome could encode proteins, whereas the rest were considered as “non-coding”. Accumulating evidence demonstrates that a variety of ncRNAs can be encapsulated and transported by exosomes, among which exosomal microRNAs (miRNAs), long non-coding RNAs (lncRNAs), and circular RNAs (circRNAs) are the most attractive subclasses in the field of bone regeneration.

#### 2.1.1 miRNAs

MiRNAs are small, highly conserved ncRNAs with ∼22 nt length (Prattichizzo et al., 2021). The biogenesis of miRNAs involves the processing of larger primary miRNAs (pri-miRNAs) into shorter pre-miRNAs, and the maturation of pre-miRNA to produce active miRNAs (Ha and Kim, 2014). MiRNAs mediate post-transcriptional gene silencing by binding to the target mRNAs 3′-untranslated region (UTR) or open reading frames (ORFs) to regulate the translational process in a wide range of physiological processes (Yang et al., 2017).

Since the first observation of exosomal miRNAs in 2007 (Valadi et al., 2007), miRNAs have become the most studied cargos in exosome. Recently, a massive number of studies have demonstrated that miRNAs in natural exosomes derived from multiple cell types can promote osteogenesis (Table 1). These studies confirmed miRNAs from exosomes of different cellular origin can enter recipient cells with the help of exosome internalization, and then regulate the expressions of genes associated with osteogenic at the translational level to regulate bone regeneration.

**TABLE 1:** The exosomal cargos involved in bone regeneration.

Cargos	Sources	Target cell	Function	References
ncRNA				
*miRNA*				
miR-23a-3p	UCMSCs	Chondrocytes BMSCs	Promoting the migration, proliferation and differentiation of chondrocytes and BMSCs	Hu et al. (2020)
miR-21	UCMSCs	EPCs	Enhancing angiogenesis	Zhang et al. (2021c)
miR-378a	M2 polarized macrophages	MSCs	Inducing osteogenic differentiation	Kang et al. (2020)
miR-100-5p	IPFP-MSCs	Chondrocytes	Enhancing the autophagy level of chondrocytes	Wu et al. (2019)
miR-335	Mature DCs	BMSCs	Promoting the proliferation and osteogenic differentiation of BMSCs	Cao et al. (2021)
miR-126	EPCs	Endothelial cells	Enhancing the proliferation, migration, and angiogenic capacity of endothelial cells	Jia et al. (2019)
miR-451a	ADSCs	Macrophages	Inhibiting inflammation and promoting the polarization of M1 macrophages to M2 macrophages	Li et al. (2022)
miR-126–5p	SCAP	HUVECs	Promoting angiogenesis	Jing et al. (2022)
miR-150–5p		MC3T3-E1	Promoting osteogenesis	
miR-29a	BMSCs	HUVECs	Promoting angiogenesis	Lu et al. (2020)
*lncRNA*				
NEAT1	Prostate cancer cells	BMSCs	Inducing osteogenic differentiation	Mo et al. (2021)
MALAT1	EPCs	Bone marrow-derived macrophages	Enhancing recruitment and differentiation of osteoclast precursors	Cui et al. (2019)
MALAT1	BMSCs	hFOB1.19	Enhancing osteoblast activity	Yang et al. (2019)
MEG-3	BMSCs	Chondrocytes	Reducing senescence and apoptosis	Jin et al. (2021)
LYRM4-AS1	BMSCs	Chondrocytes	Regulating the growth of chondrocytes	Wang et al. (2021c)
H19	BMSCs	CD31^+^ ECs and BMSCs	Promoting endothelial angiogenesis and BMSCs osteogenesis	Behera et al. (2021)
		BMSCs	Affecting osteogenic differentiation	Wang et al. (2021d)
*circRNA*				
circLPAR1	Osteogenic-induced DPSCs	DPSCs	Promoting osteogenic differentiation of the recipient DPSCs	Xie et al. (2020)
circRNA_0001236	BMSCs	BMSCs	Promoting chondrogenic differentiation	Mao et al. (2021)
circ_003564	BMSCs	Primary neurons and PC-12 cells	Attenuating inflammasome-related pyroptosis	Zhao et al. (2022)
circ-Rtn4	BMSCs	MC3T3-E1 cells	Attenuating TNF-α-induced cytotoxicity and apoptosis	Cao et al. (2020)
circ_0008542	MC3T3-E1 cells	Osteoclast	Promoting osteoclast differentiation and bone resorption	Wang et al. (2021b)
circRNA3503	SMSCs	Chondrocytes	Promoting chondrocyte renewal to alleviate the progressive loss of chondrocytes	Tao et al. (2021)
cirHmbox1	Osteoclasts	Osteoclasts and osteoblasts	Regulating osteoclasts differentiation and osteoblasts differentiation	Liu et al. (2020)
*tsRNA*				
tsRNA-10277	BMSCs	Dexamethasone-induced BMSCs	Enhancing osteogenic differentiation ability	Fang et al. (2020)
mRNA				
*TFAM*	SHED	DPSCs	Promoting osteogenic differentiation	Guo et al. (2022)
*IL-10*	M2 polarized macrophages	BMSCs	Regulating cell differentiation and bone metabolism	Chen et al. (2022)
Protein				
CD73	MSCs	Chondrocytes	Suppressing inflammation and restoring matrix homeostasis	Zhang et al. (2019)
Wnt-3a	ADSCs	Primary osteoblastic cells	Promoting the proliferation and osteogenic differentiation	Lu et al. (2017)
Mutant HIF-1α	BMSCs	BMSCs	Promoting osteogenic differentiation capacity and angiogenesis	Li et al. (2017)
		HUVECs		
BMP2	BMSCs	BMSCs	Promoting tendon bone healing in rotator cuff tear	Han et al. (2022a)

UCMSCs, umbilical cord-derived mesenchymal stem cells; IPFP-MSCs, infrapatellar fat pad mesenchymal stem cells; DCs, dendritic cells; EPCs, endothelial progenitor cells; ADSCs, adipose-derived stem cells; SCAP, stem cells from apical papilla; BMSCs, bone marrow mesenchymal stem cells; ECs, endothelial cells; DPSCs, dental pulp stem cells; SMSCs, synovium mesenchymal stem cells; SHED, stem cells from human exfoliated deciduous teeth; DPSCs, dental pulp stem cells; MSCs, mesenchymal stem cells; HUVECs, human umbilical vein endothelial cells.

#### 2.1.2 lncRNAs

As a heterogeneous group of non-protein-coding transcripts with length of greater than 200 nucleotides, lncRNAs are emerging regulators involved in diverse physiological and pathological processes (Kopp and Mendell, 2018; Nair et al., 2020). Notably, lncRNAs can be selectively packaged into exosomes (Valadi et al., 2007), which enable them as biomarkers of certain disease. For instance, the expressions of lncRNAs in serum exosomes from persons with or without osteoporosis showed significant differences (Teng et al., 2020).

Beyond as molecular markers, lncRNAs can sponge miRNAs and regulate the expression of downstream genes, called competing endogenous RNA (ceRNA) mechanism (Salmena et al., 2011). Accumulating evidence showed that lncRNAs from multiple cells-derived exosomes can enter the receptor cells and have the potential to regulate bone regeneration (Table 1).

#### 2.1.3 circRNAs

CircRNA, a special subclass of lncRNAs with a circular structure, has recently gained interest because of their extraordinary stability, much longer half-life and diverse biological functions (Jeck and Sharpless, 2014; Liu and Chen, 2022). CircRNAs can be selectively packaged into exosomes similar to lncRNAs (Ma et al., 2021). Additionally, exosomal circRNA also has the potential to regulate gene expression by ceRNA mechanism (Zhi et al., 2021; Du et al., 2022). Number of studies have revealed the regulatory function of exosomal circRNA in bone regeneration (Table 1). Interestingly, the effect of exosomal circRNAs in regulating bone regeneration, it seems, is a double-edged sword. For example, Zhi et al. (2021) reported that serum exosomal hsa_circ_0006859 was upregulated in patients with osteoporosis, and suppressed osteogenesis and promoted adipogenesis. Therefore, the regulatory functions of exosomal circRNAs are still unclear, which needs further studies.

#### 2.1.4 tsRNAs

Transfer RNA (tRNA)-derived small RNA (tsRNA) is a class of small ncRNAs generated from precursor or mature tRNAs, which has recently received considerable attention (Zhu et al., 2018; Zhu et al., 2019). With the deepening of research, tsRNAs have been reported to regulate stem cell maintenance (Blanco et al., 2016), cancer (Balatti et al., 2017), viral infection (Nunes et al., 2020), neurological diseases (Zhang et al., 2020), epigenetic inheritance (Zhang Y. et al., 2018), and symbiosis (Ren et al., 2019). The mechanisms of action of tsRNAs include playing as mimicry/replacement of tRNAs with sequence/structure effects, associating with ribonucleoproteins and binding to the target genes like miRNAs (Chen et al., 2021). Although the function of exosomal tsRNAs is an emerging field with a paucity of research, Fang et al. (2020) explored the osteogenic effect of exosomal tsRNA (Table 1). They found tsRNA-10277 in the exosome derived from BMSCs could enhance osteogenic differentiation ability of dexamethasone-induced BMSCs.

#### 2.2 mRNAs

As the Central Dogma of molecular biology presented mRNA as the fundamental ingredient in genetic translational machinery (Crick, 1970), it seemed that transferring mRNA *via* exosomes to affect the biological processes of recipient cells would be a more simple and efficient method compared with transferring ncRNAs. However, there has been remarkably little work about exosomal mRNA. This is probably because miRNAs and lncRNAs are the vast majority of exosomal RNAs (Hergenreider et al., 2012; Zhang et al., 2015; Zhang et al., 2017), and exosomal mRNAs were classically thought to be in the form of fragments, but not their intact forms (Valadi et al., 2007; Wei et al., 2017). With further research, it was estimated that on average, one intact mRNA can be found within every 1,000 exosomes produced endogenously without external stimulation (Yang Z. et al., 2020). Therefore, it is essential to confirm the integrity, high expression and regulatory function of mRNAs in the research based on exosomal mRNAs. In recent research, the regulatory function of exosomal mRNA in bone regeneration have been revealed (Table 1). These studies showed exosomal mRNAs also could be a useful tool to aid the healing of bone defects, as long as improving the loading efficiency of intrinsically encapsulate transcribed mRNA into secreted exosomes.

#### 2.3 Protein

A variety of proteins have been observed in exosomes, including cytoskeletal proteins, tetraspanins (CD9, CD63, CD81, and CD82), ESCRT-associated components (Alix and TSG101), heat shock proteins (HSP60, HSP70, and HSP90), antigen presentation proteins (MHC I and MHC II), and integrins (Kalluri and LeBleu, 2020; Zhu et al., 2020). As the main executor of life activities, proteins are not only the markers of exosomes but also endow exosomes with many biofunctions including regulating bone regeneration (Table 1).

Despite above research drawn inspiring conclusions, the controversy about the function of exosomal protein in bone regeneration persists. Take BMP2, an important regulator of osteogenesis, as an example. Han L. et al. (2022) reported that BMP2 in BMSC-derived exosomes could promote tendon bone healing in rotator cuff tear by activating Smad/RUNX2 signaling pathway. Conversely, in another study, exosomes derived from MSCs overexpressing *BMP2* did not contain BMP2 protein, and the function of promoting bone regeneration was possibly due to the changes of exosomal miRNA composition (Huang et al., 2020). Additionally, Furuta et al. (2016) found MSC exosomes could promote mice fracture healing, but the levels of SDF-1, MCP-1, and MCP-3, essential factors in the initial phase of fracture healing (Kitaori et al., 2009; Toupadakis et al., 2012; Ando et al., 2014; Ishikawa et al., 2014), in MSC exosomes were significantly lower, suggesting that bone regeneration may be mediated by other exosome components (such as miRNAs) but not exosomal proteins. The controversy above suggests that the mechanisms by which exosomal proteins work may be complex and remain to be determined.

To sum up, the function of various exosomal cargos makes exosomes have the ability to regulate bone regeneration in different ways. Predictably, more and more exosomal cargos would be revealed to function in bone regeneration by conventional or novel mechanism in the near future. Meanwhile, the explorations of mechanism inspired investigators to design engineering exosomes for bone regeneration by modifying the exosomal cargos, which will be discussed in the next section.

## 3 Strategies of engineering exosomes for bone defect repair

Although numerous exosomal cargos have been revealed to function in promoting osteogenic differentiation in the past decade, the clinical application of exosome in bone regeneration is still facing major challenges. The reason may be the low exosome yield, low content of functional exosomal cargos and low targeting efficiency of native exosomes (Song et al., 2022).

To improve the yield of exosomes, it is necessary to simplify the exosome extraction procedure. Until now, six classes of exosome separation strategies have been reported, including ultra-speed centrifugation, ultrafiltration, immunoaffinity capture, charge neutralization-based polymer precipitation, size-exclusion chromatograph, and microfluidic techniques, with unique sets of advantages and disadvantages for each technique (Yang D. et al., 2020). These rapid development in separation technology has in a large extent solved the problem of exosome isolation.

**FIGURE 2:**
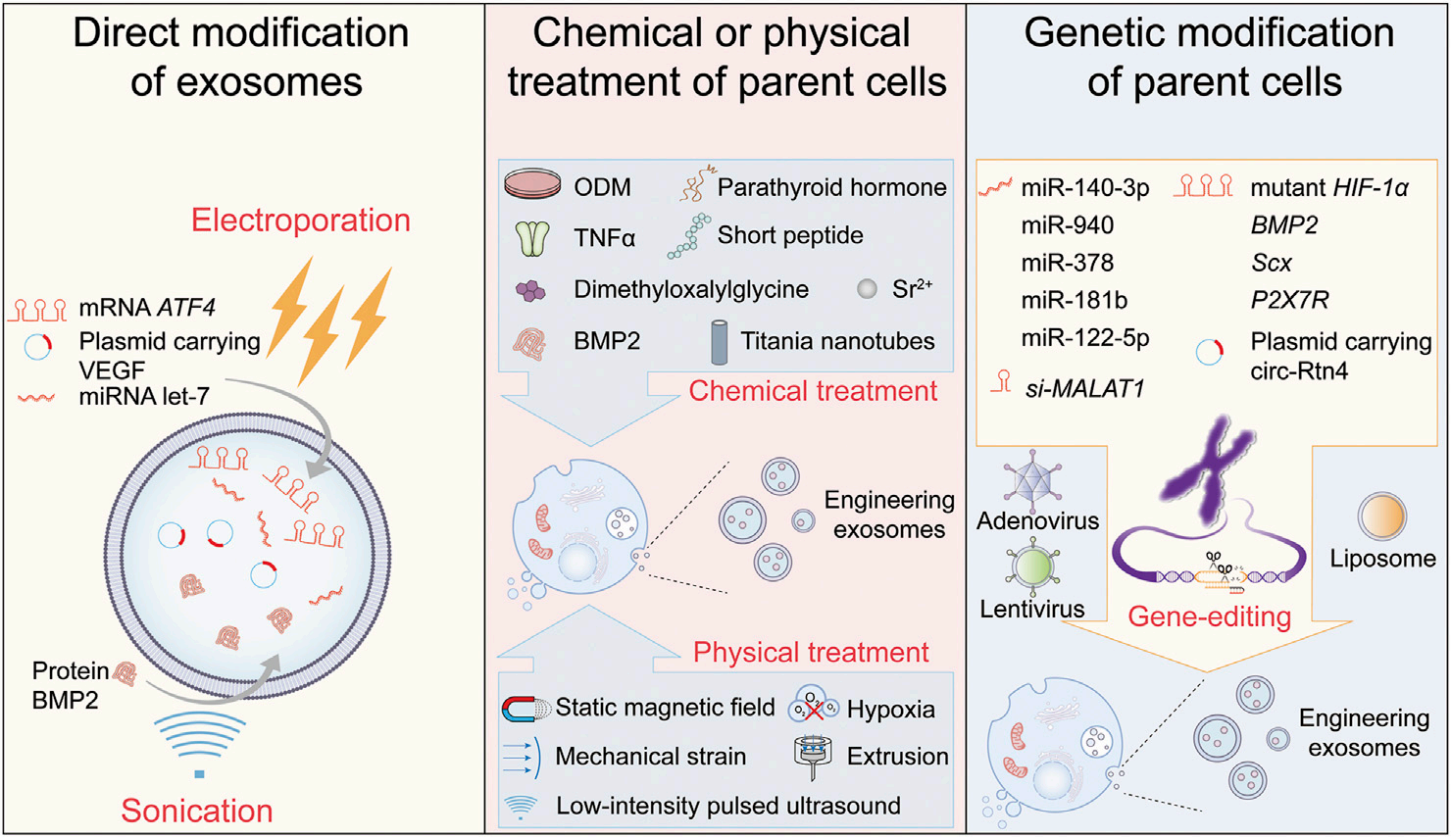
Three strategies of engineering exosomes for bone regeneration. ODM: osteogenic differentiation medium.

In order to enrich the exosomal cargo and increase exosome targeting efficiency, engineering exosome is rapidly expanding in the past decade. Engineering exosomes are the exosomes created through changing parent cells or directly on exosomes by biochemical or physical treatment (Kojima et al., 2018; Yerneni et al., 2019). In this section, we summarized the three strategies of engineering exosomes for bone regeneration (Figure 2): 1) direct modification of exosomes, 2) chemical or physical treatment of parent cells, and 3) genetic modification of parent cells.

### 3.1 Direct modification of exosomes

The direct modification of exosomes means decorating the surface proteins to improve the targeting ability of exosomes; or embellishing exosomal cargos or exogenous bioactive molecules to enhance the regulatory function through chemical methods (conjugation of peptides to exosomal surface (Gao X. et al., 2018)) or physical methods (electroporation (Tian et al., 2014) or sonication (Wang P. et al., 2019)) directly. This strategy has been extensively used to enhance the targeting ability and/or deliver specific cargo to the lesion region in numerous diseases, such as cancers (Gilligan and Dwyer, 2017; Zhang and Yu, 2019; Zhou et al., 2021), acute lung injury/acute respiratory distress syndrome (Zoulikha et al., 2022), inflammatory bowel disease (Ocansey et al., 2020) and Alzheimer’s disease (Alvarez-Erviti et al., 2011).

In bone regeneration, several studies have revealed the enhanced function of exosomes modified by electroporation and sonication. For example, Wang et al. (2021e) used electroporation to introduce activating transcription factor 4 (*ATF4*) mRNA into mice serum exosomes, and found the *ATF4*-overloading exosomes could promote chondrocyte autophagy and inhibit chondrocyte apoptosis, which in turn protected cartilage and alleviated osteoarthritic progression. Zha et al. (2021) encapsulated plasmid carrying the vascular endothelial growth factor (*VEGF*) into exosomes *via* electroporation, and the gene-activated engineering exosomes could effectively induce the bulk of vascularized bone regeneration. Choi et al. (2019) inactivated pre-osteoblast exosomal let-7, a critical miRNA regulating osteogenesis regulation, by transfecting let-7 inhibitor into exosomes *via* electroporation, and found these exosomes lost the ability to recover osteogenic differentiation, which confirmed the availability of direct modification of exosomes strategy from the opposite. Additionally, although data are scarce, sonication is another method to load hydrophilic molecules into exosomes, which is considered much more efficient than electroporation (Kim et al., 2016). In several studies, the mixture of BMP2 protein and exosomes was sonicated on ice to construct BMP2-loaded exosomes (Haney et al., 2015; Yerneni et al., 2021; Yerneni et al., 2022), and these engineering exosomes could enhance the osteogenic potential of MC3T3-E1 cells (Yerneni et al., 2022).

Direct modification of exosomes seems a simple and useful approach to obtain engineering exosomes, but the application of this strategy is still facing several challenges. The loading efficiency of electroporation is largely suppressed when transferring oligonucleotides with more than 750 bp length into exosomes (Lamichhane et al., 2015). Another important point to consider is that sonication is reported to be the most damaging technique for exosomal membrane integrity (Donoso-Quezada et al., 2020). Besides, the size and zeta potential were reported to affect the efficiency of exosome internalization (Caponnetto et al., 2017; Patel et al., 2019), which should be taken into consideration in the further research. Therefore, when using this strategy to product engineering exosomes, it must be carefully designed to increase loading and internalization efficiency and avoid exosome rupture.

### 3.2 Chemical or physical treatment of parent cells

Directly treating parent cells with chemical or physical factors is an available strategy for engineering exosomes. As originated from parent cells, the characteristics of exosomes will be reflected by the physiological and biochemical alterations of parent cells. Numerous studies have confirmed that the preconditioning of stem cells *via* hypoxia, pharmacological agents, chemical agents, trophic factors, cytokines, and physical factors could improve stem cells’ function *in vitro* and *in vivo* (Liu et al., 2011; Ferrer et al., 2013; Yang et al., 2016; Kheirandish et al., 2017; Yin et al., 2017; Hu and Li, 2018).

Chemical agents and metal ions are the two main treatment modalities of producing engineering exosomes by chemical treatment. Culturing parent cells in the osteogenic differentiation medium (ODM) is the most common method. Liu A. et al. (2021) isolated exosomes from BMSCs after osteoinductive culturing and found these engineering exosomes enhanced the bone forming capacity and induced rapid initiation of bone regeneration. In other research, umbilical cord mesenchymal stem cells (Ge and Wang, 2021) and dental pulp stem cells (Xie et al., 2020) were cultured in the ODM to produce engineering exosomes, which could enhance osteogenesis. Besides the ODM, many other chemical agents, TNF-α (Lu et al., 2017), short peptide (Zhao W. et al., 2021), parathyroid hormone (Shao et al., 2022), dimethyloxalylglycine (Liang et al., 2019) and BMP2 (Wei et al., 2019), were also used to produce engineering exosomes for bone defect repair. The metal ions treatment of parent cells can also endow exosomes with the ability to enhance bone regeneration. The exosomes derived from BMSCs stimulated by strontium-substituted calcium silicate ceramics could regulate osteogenesis and angiogenesis of human umbilical vein endothelial cells (Liu et al., 2021c). Similarly, macrophage-derived exosomes upon stimulation with titania nanotubes simultaneously enhanced osteogenesis and angiogenesis (Wang et al., 2022d).

Moreover, various physical modifications of parent cells also could yield engineering exosomes. Wu et al. (2021a) collected exosomes from BMSCs stimulated by magnetic nanoparticles and a static magnetic field and found these exosomes could improve osteogenesis and angiogenesis. As oxygen concentration plays a crucial role in proliferation (Silván et al., 2009), Shen et al. (2022) found exosomes derived from hypoxia preconditioned MSCs promote cartilage regeneration *via* the miR-205–5p/PTEN/AKT pathway. The mechanical force is an essential factor to regulate the differentiation of stem cells (Halder et al., 2012). Lv et al. (2020) found exosomes derived from osteocyte induced by mechanical strain could promote the proliferation and osteogenic differentiation of human periodontal ligament stem cell. Low yield is one of the main challenges for the application of engineering exosomes. To overcome this, Fan et al. (2020) employed an extrusion approach to amass exosome mimetics (EMs) from human MSCs, and the EMs demonstrated robust bone regeneration. In other studies, low-intensity pulsed ultrasound not only promoted BMSC-exosome release, but enhances the effects of BMSC-exosomes on cartilage regeneration in osteoarthritis (Liao et al., 2021; Xia et al., 2022).

According to above research, chemical or physical treatment of parent cells indeed is an effective strategy to produce engineering exosomes for bone regeneration. It is worthwhile to mention that the function of the engineering exosomes produced by this strategy still relies on the exosomal cargos in substantially all these studies. Therefore, modifying the nucleic acids of parent cells to produce engineering exosomes with bioactive cargos seems another ideal strategy, which will be elaborated on below.

### 3.3 Genetic modification of parent cells

With advances in molecular biological techniques, gene-editing has become one of the most commonly used methodologies in molecular research. Consequently, engineering exosomes with more or totally new bioactive molecules can be performed by editing certain genes in parent cells. As described previously, cargos are the basis of exosomes function, and numerous exosomal cargos (mRNAs, miRNAs, lncRNAs, circRNAs and proteins) have been confirmed to promote bone regeneration, which enlightened researchers to produce engineering exosomes by genetic modification of the parent cells.

As the most extensively studied exosomal cargos, miRNAs with the function of promoting bone regeneration received tremendous attention, and a vast variety of studies have attempted to enhance the biofunction of exosomes by gene-editing of parent cells’ miRNAs. Wang N. et al. (2022) transfected BMSCs with lentivirus to obtain exosomes overexpressing miR-140–3p, and found these exosomes promoted bone defect remodeling. A lentiviral infection system was also used to overexpress miR-940 in MDA-MB-231 cells to attain engineering exosomes, which could promote the osteogenic differentiation of human MSCs (Hashimoto et al., 2018). Transferring parent cells with miRNAs by Lipofectamine reagent is another genetic modification method. By this way, exosomes overexpressing miR-378 (Nan et al., 2021), miR-181b (Liu W. et al., 2021) and miR-122–5p (Liao et al., 2019) have been demonstrated to promote osteogenic differentiation.

The mRNA is also an important target for this strategy. Li et al. (2017) transfected adenovirus carrying mutant *HIF-1α* into BMSCs, and found the mutant protein was highly expressed in BMSCs exosomes, which markedly accelerated the bone regeneration and angiogenesis. Interestingly, in several other studies, exosomes derived from parent cells with gene-editing of *BMP2* (Huang et al., 2020), *Scx* (Feng et al., 2021) and *P2X7R* (Xu et al., 2020) performed the enhanced osteogenic ability. However, this modulating function was due to the changes of exosomal miRNA rather than the transfection of these genes. This might be due to two reasons: firstly, the cellular components are selectively packaged into exosomes to be exosomal cargos (Ma et al., 2021), and gene-editing of certain genes may not inevitably result in their expression change in exosome; secondly, miRNAs are the most abundant exosomal cargos, which may be more sensitive to the gene-editing modification.

Several studies revealed the effect of genetic modification on other exosomal bioactive molecules (lncRNAs and circRNAs). Cui et al. (2019) inhibited lncRNA-MALAT1 expression in endothelial progenitor cells-derived exosomes by transfecting lncRNA-MALAT1-targeting siRNA, which disrupted bone regeneration. Cao et al. (2020) subcloned the full sequence of circ-Rtn4 into the pcDNA-3.1 vector and transfected the vector into BMSCs using Lipofectamine 2000 to overexpress exosomal circ-Rtn4. Nevertheless, to date, the research in this field has remained limited for the technical reason. Take upregulating circRNAs as an example, it is difficult to deplete or generate the circular form without affecting the linear counterpart of circRNA (Nielsen et al., 2022). In addition, low cyclization efficacy and accuracy also limited the modification of circRNAs by gene-editing. Therefore, investigation into novel and high-efficiency genetic modification technologies is required to combat these problems.

## 4 Properties of exosome-integrated biomaterials essential for bone defect repair

Although the strategies of engineering exosomes could enhance exosome yield and biofunction, exosomes used for clinical bone defect treatment are still limited (van der Meel et al., 2014; Lener et al., 2015). Currently, the major modes of exosome application are direct injection or carrier loading, which is mainly aimed at systemic diseases, such as osteoporosis (Song et al., 2019), hematological malignancies (De Luca et al., 2017), and myocardial ischemia-reperfusion injury (Zhao et al., 2019). Nevertheless, it has been reported that no significant effect was observed with free exosomes treatment by direct injection, because of its rapid excretion from the site of application (Zhang Z. et al., 2018; Wang C. et al., 2019; Xing et al., 2022), suggeting the medand for exosome-integrated biomaterials. Currently, more and more biomaterials have been designed and applied in bone regeneration (Cui et al., 2020; Zhao D. et al., 2021; Zhu et al., 2022a; Zhu et al., 2022b; Wang et al., 2022c; Zhang et al., 2022). Therefore, the selection of available biomaterials with appropriate stability and integrity to load and release exosomes at the bone defect site to increase their retention and stability may be necessary for bone regeneration.

**FIGURE 3:**
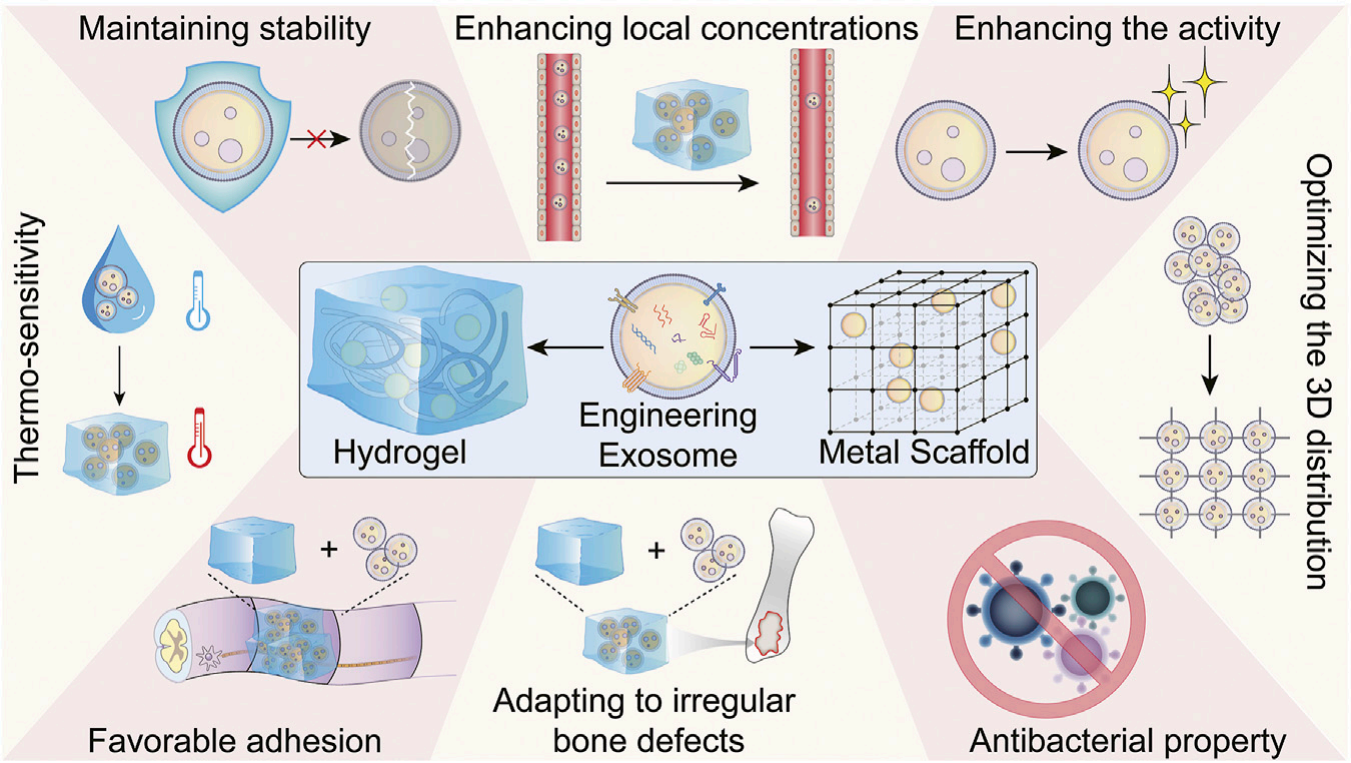
The properties of biomaterial (hydrogel and metal scaffold) help exosomes to promote bone regeneration.

Several excellent and informative reviews have addressed the types, synthetic procedure and/or encapsulation approaches of biomaterials used to carry exosomes (Riau et al., 2019; Pishavar et al., 2021; Wang D. et al., 2022; Sun et al., 2022). Instead, we propose to streamline the properties of biomaterial to dissect how and by what mechanisms the biomaterials help exosomes to promote bone regeneration (Figure 3; Table 2). By summarizing the previous studies, we expected to represent a promising strategy for the use of engineering exosomes in combination with biomaterials for clinical bone regeneration.

**TABLE 2:** The properties of exosome-integrated biomaterials for bone defect repair.

Properties	Biomaterials	References
Maintaining exosomes stability	Nanocomposite hydrogels	Li et al. (2021b)
Enhancing local concentrations of exosomes	Acellular extracellular matrix hydrogel	Xing et al. (2021)
	Injectable hyaluronic acid hydrogel	Zhang et al. (2021c)
	Gelatin methacrylate/nanoclay hydrogel	Hu et al. (2020)
	3D matrix hydrogels	Holkar et al. (2021)
	Injectable thermosensitive hydrogel	Ma et al. (2022)
Enhancing exosomes activity	3D matrix hydrogels	Yu et al. (2022b)
	Alginate hydrogel	Holkar et al. (2021)
Optimizing the 3D distribution	ABM/P-15 CMC-hydrogel	Matos et al. (2012)
	3D-printed porous bone scaffolds	Zha et al. (2021)
Antibacterial property	Food-grade probiotic-modified implant	Tan et al. (2020a)
	Multifunctional HA hydrogel	Liu et al. (2022)
		Yu et al. (2022a)
	Natural polymer HA hydrogel	Mi et al. (2022)
	Chitosan hydrogel	Shen et al. (2020)
Adapting to irregular bone defects	Injectable thermosensitive hydrogel	Xing et al. (2021)
	PDLLA-PEG-PDLLA triblock copolymer gels	Tao et al. (2021)
	Chitosan hydrogel	Fan et al. (2020)
		Wu et al. (2021b)
	Nanocomposite hydrogel based on gelatin and Laponite	Liu et al. (2021b)
	PG/TCP	Zhang et al. (2021a)
	HA hydrogel	Yu et al. (2022a)
	Alginate	Huang et al. (2020)
		Holkar et al. (2021)
	Gel-ADH	Lin et al. (2022)
	Silk fibroin	Shen et al. (2022)
	Hyaluronic acid	Sang et al. (2022)
		Yang et al. (2020b)
	SIS-CA hydrogel	Ma et al. (2022)
Favorable adhesion	Crosslinked network of alginate-dopamine, chondroitin sulfate, and regenerated silk fibroin	Zhang et al. (2021b)
	HA hydrogel modified with the PPFLMLLKGSTR peptide	Li et al. (2020)
Thermo-sensitivity	SIS-CA hydrogel	Ma et al. (2022)

ABM/P-15, CMC-hydrogel: bovine-derived mineral bound to a P-15 carboxymethyl cellulose-hydrogel; HA, hyaluronic acid; PDLLA-PEG-PDLLA, poly (D, l-lactide)-b-poly (ethylene glycol)-b-poly (D, l-lactide); PG/TCP, poly ethylene glycol maleate citrate with β-TCP; Gel-ADH, hydrazide grafted gelatin; SIS-CA, small intestinal submucosa with propionic acid.

### 4.1 Maintaining the exosome stability

The first consideration is how to maintain the stability of exosomes. Despite the bilayer membrane structures making exosomes resist degradation to some extent, exosomes are unstable and maintain for less 48 h at room temperature (Chew et al., 2019). The time will be even shorter at 37°C, at which exposed functional substances (proteins and RNA) will be rapidly degraded and metabolized. In fact, stability is an important but often overlooked point in the research of biomaterials loading exosomes, which should be given sufficient attention in the further. Hydrogel encapsulated exosomes was reported to protect them without degradation and supply therapeutic effects with persistent exosomes delivery (Riau et al., 2019). Li et al. (2021b) used the gelatin and laponite to prepare nanocomposite hydrogels as a carrier for exosomes to extend the time of BMSC-exosomes in the periodontal pocket and enhance their osteoinductive function.

### 4.2 Enhancing local concentrations of exosomes

The therapeutic effect of exosomes depends strongly on the local concentrations. However, it is demanding to produce exosomes in large quantities with high quality and purity, making clinical applications of exosomes more expensive. Additionally, free exosomes diffused out from the defect rapidly, resulting in no exertion of exosomal cargo activity (Riau et al., 2019). According to the research of Lai et al. (2012), biodistribution proceeds in the stages of liver and lungs for 30 min after direct injection of exosomes, and exosomes are removed within 1 h–6 h after administration *via* liver and kidney treatment. Thus, much research was performed with the aim of enhancing the retention and sustained-releasing of exosomes at the defect site. Xing et al. (2021) synthesized an acellular extracellular matrix hydrogel coupled with adipose-derived mesenchymal stem cell exosomes to regulate the intervertebral disc microenvironment for the treatment of intervertebral disc degeneration. The decomposition of the hydrogel was slowed down, allowing exosomes to remain in the disc for up to 28 days Zhang Y. et al. (2021) fabricated an injectable hyaluronic acid hydrogel encapsulated with umbilical MSC-derived exosomes through three-dimensional (3D) printing technology, and the hydrogel showed good sustained-releasing features in the rat critical-size cranial defect model. Hu et al. (2020) fabricated Gelatin methacrylate/nanoclay hydrogel for sustained release of exosomes. The hydrogels with a 3D matrix prevent the dispersion of exosomes and maintained their local concentration, which enable the controlled release of exosomes at bone defect sites (Holkar et al., 2021). In another study, exosomes were incorporated into an injectable thermosensitive hydrogel by constructing fusion peptides, which also enhanced the retention of exosomes and improved the biological activity of exosomes (Ma et al., 2022). Generally, the consensus has been achieved that it is essential to enhance local exosome concentrations at the bone defect site, and the biomaterials loading exosomes should possess the property of enhancing the retention and sustained-releasing of exosomes.

### 4.3 Enhancing exosomes activity

The hydrogel with 3D microenvironment can enhance exosome activity and affect the interaction of integrin membrane protein between cells and the cell matrix, which promotes cell proliferation and differentiation in a bone regeneration environment. Yu W. et al. (2022) encapsulated exosomes derived from periodontal ligament stem cells into a hydrogel with 3D microenvironment, which enhanced osteoinductive ability and significantly promoted bone defect repair in rats. Another study demonstrated alginate hydrogels combined with exosomes promoted osteogenesis by increasing cell-exosomes interactions, cell aggregation, and long-term viability (Holkar et al., 2021).

### 4.4 Optimizing the 3D distribution of exosomes

Biomaterials with a highly porous and 3D structure mimic the porosity, pore size, and interconnectedness of native bone ideally. In bone defects repair, bioactive materials with good mechanical properties not only provide temporary mechanical support for the bone at the implant site, but also modulate extracellular matrix formation, facilitate better cell-cell and cell-matrix interactions, retain the cell morphology, provide mechanical stimulations, and support cell growth and exosomes secretion, the features which are akin to *in vivo* systems (Tibbitt and Anseth, 2009). Matos et al. (2012) showed that the lyophilized biomaterials created a more homogenous interparticle spacing, allowed a more suitable particle distribution and stabilization, then promoting a faster bone regeneration with relevant clinical benefits. Similarly, biocompatible 3D porous biomaterials ensured a uniform spacing and stable distribution of MSC-exosomes compared with compacted materials (Zha et al., 2021).

### 4.5 Antibacterial property

During bone defect healing, the bacterial infection is one of the risk factors (Blair et al., 2015). Therefore, antibacterial property of biomaterials should also be taken into consideration. Tan L. et al. (2020) developed a food-grade probiotic-modified implant to prevent methicillin-resistant *Staphylococcus aureus* infection and accelerated bone integration. Liu et al. (2022) designed a multifunctional hyaluronic acid (HA) hydrogel with antibacterial property. Then, this group loaded plasma exosomes to this hydrogel for promoting infected fracture healing (Yu C. et al., 2022). Mi et al. (2022) combined engineered exosomes and a natural polymer HA hydrogel, which performed an anti-inflammatory and antibacterial function on fracture repair acceleration. As a cationic natural polymer biomaterial, chitosan has anti-microbial property (Dai et al., 2011), and many reports have shown cationic loaded engineering exosomes could promote bone regeneration (Fan et al., 2020; Shen et al., 2020; Wang et al., 2020; Wu et al., 2021b; Bahar et al., 2022; Nikhil and Kumar, 2022), although some of them did not look at the antibacterial property. The biomaterials with antibacterial property have been designed in some studies, but these materials are still rarely used for bone defect repair, which merits further investigation.

### 4.6 Adapting to irregular bone defects

Clinical bone defects caused by trauma, neoplasia, infection or corrective osteotomies are always irregular. Hence, biomaterials should have the injectable property to fill irregular defects and promote *in situ* bone tissue regeneration. Xing et al. (2021) constructed an injectable thermosensitive hydrogel system *via* a coordinative crossing of ADSC-derived exosomes and acellular extracellular matrix hydrogels to effectively protect nucleus pulposus cells from pyroptosis after intervertebral disc degeneration. By taking advantage of injectable, reversible, and thermosensitive abilities, Tao et al. (2021) used PDLLA-PEG-PDLLA triblock copolymer gels as a carrier of synovium mesenchymal stem cells-derived exosomes for intra-articular injection to prevent osteoarthritis progression. Additionally, multiple biomaterials, including chitosan hydrogel (Fan et al., 2020; Wu et al., 2021b), nanocomposite hydrogel (based on gelatin and Laponite) (Liu et al., 2021b), PG/TCP (PEGMC with β-TCP) (Zhang B. et al., 2021), HA hydrogel (Yu C. et al., 2022), alginate (Huang et al., 2020; Holkar et al., 2021), Gel-ADH (hydrazide grafted gelatin) (Lin et al., 2022), silk fibroin (Shen et al., 2022), hyaluronic acid (Yang S. et al., 2020; Sang et al., 2022) and SIS-CA (small intestinal submucosa (SIS) with propionic acid (CA)) hydrogel (Ma et al., 2022) were reported to adapt irregular bone defects and were used to carry exosomes for skeletal regeneration. Generally, although local injection therapy is not suitable for certain types of bone regeneration, such as spinal cord repair (Han M. et al., 2022), injectable biomaterials loading engineering exosomes have been used extensively to repair irregular bone defects.

### 4.7 Other properties

In a moist environment, the favorable adhesion of biomaterials is essential for *in situ* bone regeneration (Hasani-Sadrabadi et al., 2020; Li et al., 2020; Zhang FX. et al., 2021). Inspired by mussel materials, which exhibit underwater robust adhesion (Gao Z. et al., 2018), Zhang and the colleague (2021b) prepared a hydrogel with high bonding strength to the wet surface using a crosslinked network of alginate-dopamine, chondroitin sulfate, and regenerated silk fibroin, which promote cartilage defect regeneration by combining with BMSCs-exosomes. Peptide-modification is another strategy for enhancing biomaterial adhesion. Li et al. (2020) prepared a biomaterial with high adhesion by modifying HA hydrogel with the PPFLMLLKGSTR peptide, which could locally deliver human placenta amniotic membrane mesenchymal stem cell-derived exosomes in spinal cord tissue.

Biomaterials with thermo-sensitivity are also of particular concern for their property, changing between a liquid state and a solid-state based on the ambient temperature (López-Noriega et al., 2014; Ni et al., 2014). Several thermo-sensitive biomaterials have been used in bone defect repair (Fu et al., 2012; Kim et al., 2018; Yu et al., 2020; Wang QS. et al., 2021). Further, Ma et al. (2022) designed a novel thermo-sensitive biomaterial by loading BMSCs-exosomes with SIS-CA hydrogel to regulate bone regeneration.

Collectively, the bio-functional materials not only provide a scaffold or carrier for engineering exosomes, but also play an essential role by their own variety properties. Along with major advancements in chemical engineering techniques, more and more novel biomaterials with various properties have been synthesized for bone regeneration. In the future, selection of appropriate biomaterials to integrate engineering exosomes should be one of the leading focuses of bone defect repair.

## 5 Conclusion and perspective

Coexistence of challenges and opportunities have greatly stimulated the study of engineering exosomes for bone regeneration in the past 10 years. In the present review, we mainly addressed the molecular basis of exosomal cargos, the strategies of engineering exosomes and the properties of exosome-integrated biomaterials required for bone regeneration. The research about engineering exosomes for bone defect repair is undeniably in its infancy. The rapid development of engineering exosomes is impeded by several key challenges, especially the consistency of exosomes production. As a result, these difficulties inspired the development of new and cutting-edge approaches, often distinct from those in the conventional study of cells, to address both exosome production and function. Refining the isolation, purification and storage techniques of exosomes may be an effective means of improving the consistency of exosomes production (Colao et al., 2018; Zeng et al., 2022). Additionally, excellent biomaterials emerged continuously, which greatly promoted research self-renewal. The effective combination of engineering exosomes and biomaterials will be greater than the sum of their parts and exhibit synergy effects in bone regeneration. We optimistically foresee that novel biomaterials will be constructed and more sophisticated engineering exosomes will be implemented for bone tissue regeneration. This huge progress is sure to benefit both biomedical research and therapeutic modalities in the field of bone regeneration.
